# Mechanism of focal cerebral ischemic tolerance in rats with ischemic preconditioning involves MyD88- and TRIF-dependent pathways

**DOI:** 10.3892/etm.2013.1318

**Published:** 2013-09-27

**Authors:** HAN LI, MINGYUE JIN, TAO LV, JUNHONG GUAN

**Affiliations:** Department of Neurosurgery, Shengjing Hospital, China Medical University, Shenyang, Liaoning 110004, P.R. China

**Keywords:** Toll-like receptor 4, ischemic preconditioning, brain ischemic tolerance, myeloid differentiation factor 88

## Abstract

The aim of this study was to explore the involvement of Toll-like receptor 4 (TLR4) and the downstream myeloid differentiation factor 88 (MyD88)-dependent and -independent pathways in the mechanisms of cerebral ischemic tolerance. Using an improved middle cerebral artery occlusion method, we constructed a preconditioned ischemic brain model in rats. Sham and ischemia-reperfusion groups were also established. The expression levels of proteins in the MyD88/nuclear factor-κB (NF-κB) pathway (MyD88-dependent) were compared with those in the Toll/interleukin-1 receptor-domain-containing adaptor-inducing interferon-β (TRIF)/interferon regulatory factor-3 (IRF-3) pathway (MyD88-independent) by western blot analysis. NF-κB and IRF-3 protein expression levels within cells were determined by immunofluorescence staining of frozen tissue sections. Western blot analysis showed a downregulation of MyD88 protein expression in the brain tissue of ischemic preconditioned rats; however, NF-κB, TRIF and IRF-3 protein expression levels were upregulated. Immunofluorescence staining showed that NF-κB protein was mainly located in the cytoplasm in ischemic preconditioned rats and IRF-3 was predominantly located in the nucleus. The results indicate that changes in the two TLR4 downstream pathways are the main mechanisms involved in the development of brain ischemic tolerance with ischemic pretreatment.

## Introduction

Hypoxic-ischemic brain damage (HIBD) is irreversible; it releases cytokines and oxygen free radicals that exacerbate ischemic brain damage through inflammation and apoptosis (1). In the necrotic area and ischemic brain tissues following cerebral infarction, excessive inflammatory and immune responses are the pathophysiological basis of tissue damage (2). Toll-like receptors (TLRs) are indispensable in the inflammatory response and may recognize endogenous damage-associated molecular patterns (DAMPs) and exogenous pathogen-associated molecular patterns (PAMPs) when affiliated ligands are activated. In addition, TLRs induce nuclear factor-κB (NF-κB)-associated pro-inflammatory cytokines through a myeloid differentiation factor 88 (MyD88)-dependent pathway and produce interferon regulatory factor (IRF) through a MyD88-independent pathway to promote downstream pathways (3).

TLRs are activated when bacteria or virus-associated molecular patterns are identified, and then promote an inflammatory response mediated by macrophages, neutrophils and complement (4). It has been suggested that a small dose of a bacterial endotoxin (PAMP) may induce endotoxin tolerance through the modulation of TLRs and reduce the damage mediated by an excessive inflammatory response (5–7). Previous studies have shown that small doses of endotoxin may result in tolerance to ischemic brain injury and protective effects (8,9), suggesting that pretreatment with exogenous PAMP may induce endogenous DAMP tolerance. However, the mechanism by which brain tissues become ischemia tolerant with endogenous DAMP pretreatment remains unknown.

Transient ischemic preconditioning is commonly used for studying the role of endogenous TLR ligands and has been shown to have protective effects against ischemia-reperfusion injury in the brain (10). A previous study identified that neuroprotective functions were weaker following transient cerebral ischemia in TLR4 gene-deficient mice, suggesting that the release of endogenous TLR ligands induces cerebral protective mechanisms (11). The exact mechanisms of action of the TLRs and the downstream MyD88-dependent and -independent pathways require further study. In the present study, an ischemic preconditioning model with a line embolism blocking the middle cerebral artery was established in rats and the expression levels of MyD88, NF-κB, Toll/interleukin-1 receptor-domain-containing adaptor-inducing interferon-β (TRIF) and IRF-3 were detected at three time-points following reperfusion.

## Materials and methods

### Animal grouping

Twenty-seven male Sprague Dawley rats were purchased from the Vital River Laboratory Animal Technology Co., Ltd. (Beijing, China) and were randomly divided into three groups: The sham group (Group A) received sham treatment twice (n=3); the ischemia-reperfusion group (Group B) received one sham surgery and one ischemia-reperfusion treatment (n=12); and the ischemic preconditioning group (Group C) received one ischemic preconditioning and one ischemia-reperfusion surgery (n=12). According to different time-points of animal sacrifice, the groups were each divided into three subgroups. The subgroups were A12, A24, A48, B12, B24, B48, C12, C24 and C48, with the letter corresponding to the group name, and the number to the time following reperfusion; for example, A12 included animals from Group A sacrificed 12 h following reperfusion. The present study was approved by the Ethics Committee of Shengjing Hospital (Shenyang, China).

### Establishment of the ischemic preconditioned rat model

The rats in Group C were anaesthetized with 10% chloral hydrate (3.5 ml/kg) and the common carotid artery, external carotid artery and internal carotid artery were blunt separated. The proximal and distal ends of the common carotid artery were ligated with surgical sutures (size, 3–0), bifurcation of the internal and external carotid artery was closed with artery occlusion; an additional artery occlusion was used to close the external carotid artery. A small cut was made between the common carotid artery ligation and the vascular clamp. The line embolism was inserted into the middle cerebral artery and reperfusion was induced following 10 min of blocking. The subcutaneous tissues and skin tissues were sutured at each layer. In Groups A and B, only the common, external and internal carotid arteries were blunt separated, and the subcutaneous tissues and skin tissues were sutured at each layer. All animals were conventionally fed for two days. After these two days, the separated common, external and internal carotid arteries were identified. In Group C, the distal end and the bifurcation of the internal and external carotid arteries were closed with artery occlusion and a new surgical line replaced the old one that had been inserted two days previously. An additional artery occlusion was performed to close the external carotid artery and prevent line embolism in the external carotid artery. A small cut was made between the common carotid artery ligation and vascular clamp, a line embolism was inserted in the middle cerebral artery and reperfusion was induced for 12, 24 and 48 h following 60 min of blocking. In Group B, the ischemia-reperfusion method was the same as that in Group C. In Group A, only the common, external and internal carotid arteries were blunt separated, and the subcutaneous and skin tissues were sutured at each layer.

### Specimen grouping at 12, 24 and 48 h

In Group A, one rat was sacrificed at 12 h, one at 24 h and one at 48 h after ischemia-reperfusion, to constitute the A12, A24 and A48 subgroups, respectively. In Groups B and C, four rats were sacrificed at each of 12, 24 and 48 h after ischemia-reperfusion to constitute the B12, B24 and B48 and C12, C24 and C48 subgroups, respectively. In addition, brain tissue from each rat was collected for further experiments.

### Western blot analysis of MyD88, NF-κB, TRIF and IRF-3

Protein extracts were prepared using radioimmunoprecipitation assay (RIPA) lysis buffer and the protein concentrations were measured by the Bradford method using a BCA Protein assay kit (Pierce Biotechnology, Inc., Rockford, IL, USA). Equal quantities of protein extracts (30 μg) were separated by 12% sodium dodecyl sulfate-polyacrylamide gel electrophoresis (SDS-PAGE) and then transferred onto 0.45 μm polyvinylidene difluoride (PVDF) membranes (Millipore, Billerica, MA, USA) in wet conditions. The membranes were blocked in 1X Tris-buffered Saline and Tween 20 (TBST) containing 5% non-fat dry milk for 1 h and then incubated at 4°C overnight with rabbit anti-MyD88, -NF-κB, -TRIF and -IRF-3 antibodies (sc-179; Santa Cruz Biotechnology, Inc., Santa Cruz, CA, USA) or rabbit anti-β-actin antibody (AP0060; Bioworld Technology, Inc., St. Louis Park, MN, USA), both diluted 1:1,000 in 1X TBST containing 5% non-fat dry milk. This was followed by incubation with a secondary horseradish peroxidase-conjugated anti-rabbit antibody (BS13278; Bioworld Technology, Inc.) diluted 1:5,000 in 1X TBST containing 5% non-fat dry milk for 1 h at room temperature. Proteins were detected using an enhanced chemiluminescence system (Pierce Biotechnology, Inc.) in a dark room. Western band densities were quantified using ImageJ software, version 1.44p (National Institutes of Health; http://rsbweb.nih.gov/ij/. Accessed September 15, 2013).

### Immunofluorescence staining of NF-κB and IRF-3

Tissues were fixed in 4% paraformaldehyde for 24 h and then embedded in paraffin. Sections were prepared for hematoxylin and eosin staining. Immunohistochemical staining of formalin-fixed paraffin-embedded sections was performed. Tissue sections (4 μm thick) were dewaxed in toluene, rehydrated, permeabilized in citrate buffer (pH 6.0) and quenched with 3% H_2_O_2_ for 15 min to eliminate endogenous peroxidase activity. The sections were then incubated overnight at 4°C with a primary antibody for rabbit anti-NF-κB, anti-NF-κb-Cy3, anti-IRF-3-FITC (diluted 1:100 in PBS). After washing in phosphate-buffered saline, tissues were incubated with biotinylated goat anti-rabbit immunoglobulin G (Dako, Glostrup, Denmark) followed by treatment with peroxidase-conjugated streptavidin and staining with diaminobenzidine. Counterstaining was performed with hematoxylin. Immunostaining was evaluated by two independent observers unaware of the origin of the tissue.

### Statistical analyses

All data are expressed as the mean ± standard deviation. SPSS software, version 18.0 (SPSS, Inc., St. Louis, MO, USA) was used for statistical analysis of the data. Data between two groups was analyzed by Student’s t-test and data from several groups was evaluated with variance analysis. P<0.05 was considered to indicate a statistically significant difference.

## Results

### Downregulation of MyD88 and NF-κB protein expression by ischemic preconditioning

**Figure 1:**
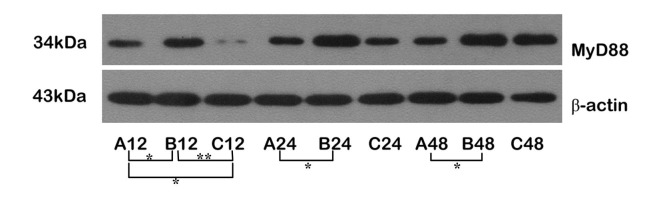
Western blot analysis of MyD88 protein expression in the sham (Group A), ischemia-reperfusion (Group B) and ischemic preconditioning groups (Group C) at 12, 24 and 48 h following reperfusion. ^**^P<0.01 and ^*^P<0.05. MyD88, myeloid differentiation factor 88.

Following transient ischemic preconditioning (Group C), the level of MyD88 protein expression was downregulated at all time-points. In the ischemia-reperfusion group without preconditioning (Group B), MyD88 protein expression was upregulated at all time-points. The MyD88 expression level of the C12 subgroup was significantly lower than that of the B12 subgroup (P<0.01) and was also significantly lower than that of A12. The MyD88 expression level of B12 was significantly higher than that of A12. These results showed that the expression level of MyD88 protein was significantly increased following 60 min of ischemia followed by reperfusion for 12 h and confirmed that transient ischemic preconditioning inhibited the expression of MyD88. We observed that MyD88 expression was upregulated in Group B, and the expression levels were significantly higher than those of Group A. In addition, MyD88 protein expression was upregulated in Group C, but at a low level. The MyD88 expression level of the C24 subgroup was lower than that in normal brain tissue (Group A), indicating that ischemic preconditioning is able to inhibit the overexpression of MyD88 (Fig. 1).

**Figure 2:**
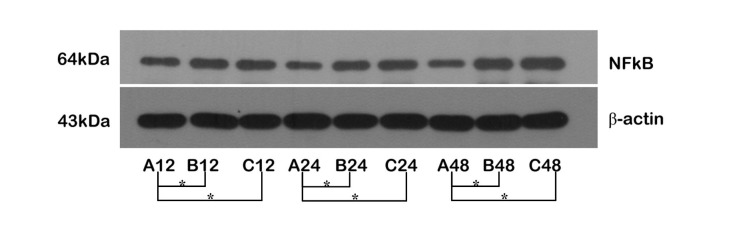
Western blot analysis of NF-κB protein expression in the sham (Group A), ischemia-reperfusion (Group B) and ischemic preconditioning groups (Group C). A12, 12 h following reperfusion in Group A.^*^P<0.05. NF-κB, nuclear factor-κB.

The expression of NF-κB was upregulated at all time-points in Groups B and C (Fig. 2); the expression of NF-κB in the C48 subgroup was marginally higher than that in the B48 subgroup, but there were no significant differences between Groups B and C. These results indicate that the expression of NF-κB was inconsistent with the expression of MyD88, suggesting the upregulation of NF-κB was regulated through additional pathways.

### Upregulation of TRIF/IRF-3 protein expression through the MyD88-independent pathway

**Figure 3:**
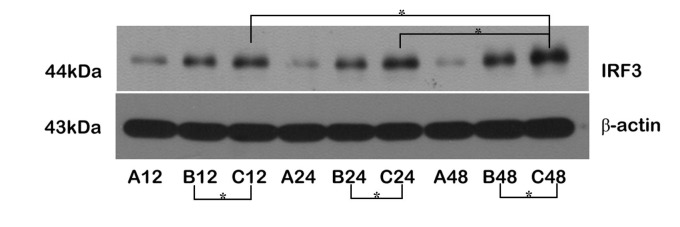
Western blot analysis of IRF-3 and protein expression in the sham (Group A), ischemia-reperfusion (Group B) and ischemic preconditioning groups (Group C) at 12, 24 and 48 h after reperfusion.^*^P<0.05. IRF-3, interferon regulating factor-3.

**Figure 4:**
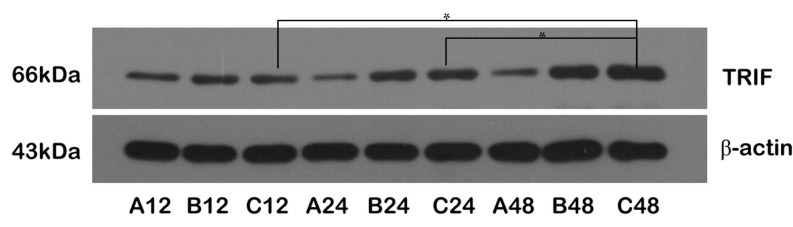
Western blot analysis of TRIF protein expression in the sham (Group A), ischemia-reperfusion (Group B) and ischemic preconditioning groups (Group C) 12, 24 and 48 h after reperfusion.^*^P<0.05. TRIF, Toll-like receptor domain-containing adaptor-inducing interferon-β.

The expression levels of the TRIF and IRF-3 proteins were upregulated following reperfusion. The expression levels of IRF-3 protein were higher in Group C than in Group B at all time-points; the expression levels of TRIF and IRF-3 were significantly increased in the C48 subgroup, but only the expression of IRF-3 was marginally increased at C12, suggesting that the expression levels of TRIF and IRF-3 were upregulated through transient ischemic preconditioning. Comparing the expression levels of TRIF and IRF-3 proteins between Groups B and C, the changes in the expression levels of IRF-3 were larger than those of TRIF, indicating that the expression of IRF-3 was regulated by TRIF during the cerebral protective process, and ischemic pretreatment may have facilitated this process, resulting in brain ischemic tolerance (Figs. 3 and 4).

### Coordination of TRIF/IRF-3 and NF-κB proteins in ischemic preconditioning

The upregulation of NF-κB and TRIF/IRF-3 protein expression showed coordination at certain time-points; in particular, NF-κB and TRIF proteins were upregulated at 12 and 24 h following reperfusion in Groups B and C. The expression levels of NF-κB, TRIF and IRF-3 were increased 48 h following reperfusion and the protein expression levels were higher in Group C than in Group B (P>0.05).

### Location of NF-κB and IRF-3 proteins

**Figure 5:**
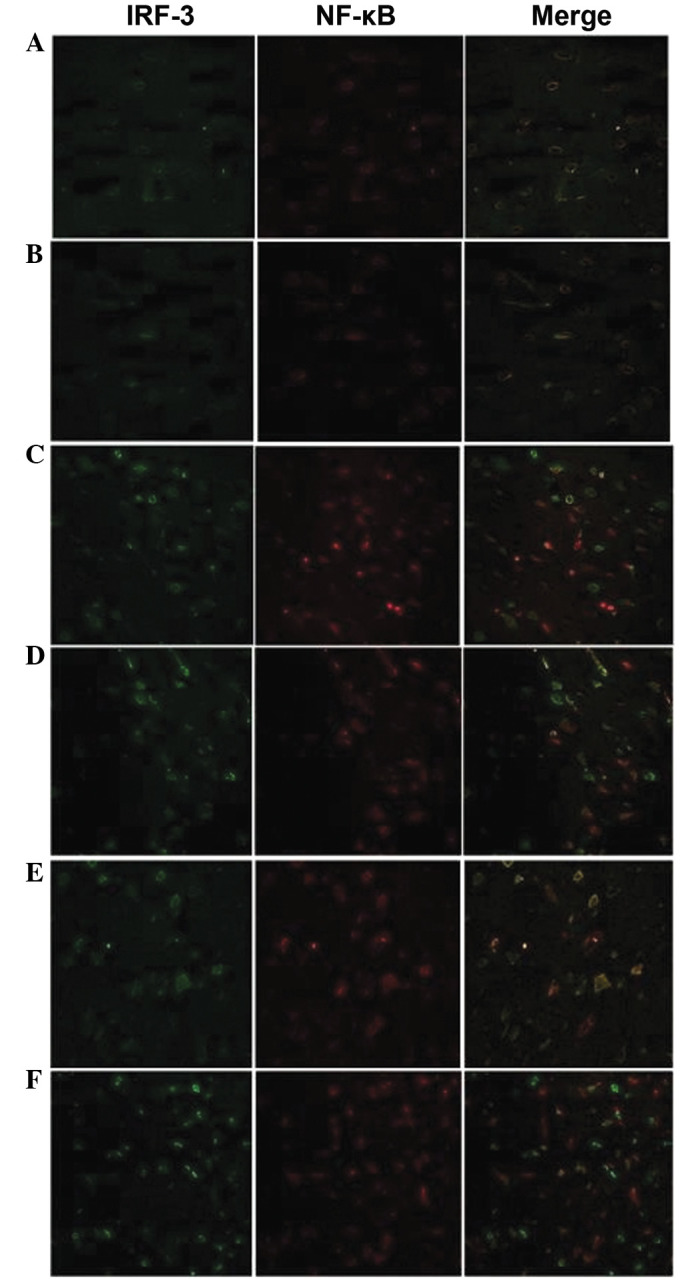
Immunoprecipitation and immunofluorescence of frozen tissue sections were observed to analyze NF-κB and IRF-3 protein expression in the sham (Group A), ischemia-reperfusion (Group B) and ischemic preconditioning groups (Group C). NF-κB shows red fluorescence when labeled with cy3, IRF-3 shows green fluorescence when labeled with FITC. Protein location in the (A) A12 and (B) A48 subgroups showed no significant differences at 12 and 48 h. Immunoprecipitation results of the (C) B24 and (D) B48 subgroups indicated expression in the cytoplasm and nucleus. Expression of NF-κB and IRF-3 proteins in the (E) C12 and (F) C48 subgroups showed the expression of IRF-3 was increased in the nucleus and the expression of NF-κB was increased in the cytoplasm. NF-κB, nuclear factor-κB; IRF-3, interferon regulating factor-3.

Without ischemic preconditioning and ischemia-reperfusion, the NF-κB and IRF-3 proteins were located in the cytoplasm and the fluorescent brightness was consistent with the results of the semi-quantitative western blot analysis (Fig. 5A and B).

The fluorescent brightness of NF-κB and IRF-3 proteins was elevated in the B12 and B24 subgroups compared with those in Group A, and the proteins, particularly NF-κB, were transferred into the nucleus. However, the distribution of NF-κB and IRF-3 proteins showed no significant changes in the B48 subgroup from those in B12 and B24, and the IRF-3 protein was mainly distributed in the nucleus (Fig. 5C and D).

Notably, the distribution and fluorescent brightness of NF-κB and IRF-3 proteins in the C12 subgroup was similar to that of the B48 subgroup and higher than that of Group A. The proteins, particularly IRF-3, were transferred into the nucleus. The NF-κB protein was transferred from the nucleus to cytoplasm in the C24 and C48 subgroups, and IRF-3 protein was mainly expressed in the nucleus. The fluorescence brightness of the IRF-3 protein was higher in the C48 subgroup than in the C12 and C24 subgroups (Fig. 5E and F).

## Discussion

A previous study showed that the ischemic tolerance of endotoxins through TLR4 was regulated by NF-κB of the MyD88-dependent pathway and IRF-3 of the MyD88-independent pathway. The study focused on the endogenous DAMPs recognized by TLR4 and the ischemic tolerance mechanisms (12).

The results of the present study identified that MyD88 protein expression was significantly inhibited following transient cerebral ischemia and the expression of MyD88 remained low even following reperfusion for 12 h. We speculate that the inhibitory effects are transient and the delayed expression of MyD88 protein may subsequently prevent the inflammatory response, which is caused by activation of the inflammatory pathway. The expression level of MyD88 protein was increased at 24 and 48 h following reperfusion in the ischemic-preconditioned group and the level of expression at 48 h was higher than that in the control group; however, the MyD88 expression level was lower than that of the ischemia-reperfusion group. It is known that the MyD88-dependent pathway induces the inflammatory response, and is associated with the proliferation and differentiation of nerve cells following injury (13). The inflammatory cytokines and anti-inflammatory cytokines may achieve a steady balance so as to repair nerve injury (14). It has been demonstrated that ischemic tolerance is induced by inflammatory cytokines, such as tumor necrosis factor-α, but inhibited by glucocorticoids (15). Therefore, the MyD88 protein was marginally reduced, but not completely inhibited following transient ischemic preconditioning; with a suitable time of ischemia-reperfusion, the appropriate inflammatory cytokines may induce ischemic tolerance.

NF-κB is the downstream cytokine of the MyD88-dependent pathway and its expression levels were inconsistent with those of MyD88. In the present study, we observed that the expression levels of NF-κB were not affected by ischemic preconditioning after 12 and 24 h of reperfusion and the expression levels were higher than those of the normal control group. The expression of the TRIF protein was upregulated following ischemic preconditioning, but there were no significant differences in TRIF expression between the ischemia-reperfusion and ischemic preconditioning groups at 12 and 24 h. At 48 h, the expression levels of TRIF, IRF-3 and NF-κB were significantly increased, and significant differences were identified between the ischemia-reperfusion and ischemic preconditioning groups. The TRIF/IRF-3 pathways is the main signaling pathway for neuroprotection with endotoxin or ischemia and hypoxia preconditioning (16,17). In the present study, we observed that the TRIF/IRF-3 expression levels were upregulated following transient ischemic preconditioning. In addition, the changes in the expression of NF-κB protein were induced by the action of the MyD88 and TRIF pathways, which suggests that the expression changes of NF-κB protein were mainly mediated by the late NF-κB activation of the TRIF pathway. The sudden elevation of the NF-κB expression levels at 48 h may have been caused by the activation of the TBK1/IKKɛ pathway (18). We also observed that NF-κB migrated from the nucleus to the cytoplasm and IRF-3 gradually transferred to the nucleus with reperfusion. Notably, in Group B, NF-κB was mainly expressed in the nucleus at 12 and 24 h, but IRF-3 was mainly expressed in the nucleus at 48 h. NF-κB is the cytokine necessary for regulating the inflammatory response, growth and differentiation. In lymphocytes, NF-κB interacts with the inhibitory protein, IκB, to maintain an inactive state; however, when IκB protein is degraded, NF-κB translocates to the nucleus to combine with genes (19). NF-κB activity was significantly inhibited by endotoxins following ischemia-reperfusion for 24 h and inhibitory proteins, such as Ship1 and Tollip, were found in the cytoplasm, which would not affect the expression of pro-inflammatory genes (17). Therefore, in our study, although the expression of NF-κB was increased, it was maintained in an inactive state in the cytoplasm and IRF-3 protein was upregulated in the nucleus to achieve cerebral protection. However, the inflammatory cytokines and pro-inflammatory cytokines were not investigated.

In conclusion, to the best of our knowledge, we conducted transient ischemic preconditioning in rats for the first time. We investigated the associated patterns of endogenous injury recognized by TLRs, and observed the expression of proteins associated with the MyD88-dependent and -independent pathways at 12, 24 and 48 h following ischemia-reperfusion. In addition, we outlined the cerebral protective mechanisms of ischemic preconditioning and provided a reference for further study. Our findings indicate a potential therapeutic approach for the treatment of hypoxic-ischemic brain damage.
